# Human-computer interaction: catalyst or drain? Dual pathways of employee creativity from a psychological resources perspective

**DOI:** 10.3389/fpsyg.2026.1793892

**Published:** 2026-08-19

**Authors:** Minling Chen, Wei Liu

**Affiliations:** School of Economics and Management, Xi’an Shiyou University, Xi'an, China

**Keywords:** burnout, employee creativity, human-computer interaction, perceived organizational support, psychological availability

## Abstract

**Purpose:**

With digital technology and AI increasingly embedded in work, human-computer interaction design has become pivotal in unlocking intelligent technologies’ creative potential for both research and practice. Most research focuses on AI’s association with employee creativity but rarely explores the psychological mechanisms linking human-computer interaction to employee creativity. This study examines the double-edged nature of this association from a psychological resources perspective.

**Design/methodology/approach:**

An online survey of employees in firms undergoing digital and intelligent transformation yielded 367 valid responses. Hypotheses were tested via structural equation modeling, regression, and bootstrap analyses, examining the association between human-computer interaction modes and employee creativity. Psychological availability and burnout served as mediators, and perceived organizational support as a moderator.

**Findings:**

Findings suggest that human-computer interaction is linked to employee creativity through both positive and negative pathways. In the positive pathway, human-computer interaction is positively associated with psychological availability, which is in turn positively associated with employee creativity. In the negative pathway, human-computer interaction is positively associated with burnout, which is negatively associated with employee creativity. Further analysis shows that perceived organizational support moderates both pathways: it is associated with a strengthening of the positive mediating effect of psychological availability and a weakening of the negative mediating effect of burnout. Thus, a moderated dual mediation model is supported.

**Originality/value:**

This study contributes to the human-computer interaction and employee creativity literature in three ways. First, it examines the psychological mechanisms linking the two, moving beyond a narrow focus on positive associations. Second, adopting a psychological resources lens, it reveals a double-edged pattern, with psychological availability and burnout serving as dual mediators. Finally, it identifies perceived organizational support as a key moderator, establishing a moderated dual-mediation model. This provides insights into leveraging human-computer interaction for creativity.

## 1 Introduction

In the context of the deepening digital transformation process, the importance of employee creativity in helping firms overcome developmental challenges and build core competitiveness is becoming increasingly evident (Acar et al., 2019). Employees’ innovative thinking and practical abilities are essential for process reform, product innovation, and service optimization. The degree of employee creativity directly affects an organization’s capacity to survive and grow sustainably in the face of market competition (Anderson et al., 2014). The rapid advancement of artificial intelligence technology and its widespread incorporation into corporate processes are significantly changing employee work patterns. AI has emerged as a crucial workplace partner, offering everything from data processing and job collaboration to decision support (Deng et al., 2025; Kamoonpuri and Sengar, 2023). Its technological value is increasingly dependent on effective collaboration with employees. Therefore, human-computer interaction—which acts as the primary conduit between employees and AI systems—has gradually come to be recognized as a critical factor affecting worker productivity and innovation performance (Xiaoman and Li, 2024). Academics have increasingly studied the connection between employee creativity and human-computer interaction recently. Empirical research has demonstrated that human-computer collaboration serves as a new source of employee creativity (Chen and Chan, 2024). In light of this, it is especially important and of practical value to investigate how human-computer interaction fosters and improves employee creativity.

As AI technology develops, human-computer interaction become increasingly frequent and seamless (Kim and Im, 2025). Accordingly, studies on human-computer interaction have become essential in several fields, such as business innovation, intelligent vehicles, and healthcare (An et al., 2024; Guo et al., 2025; Zhang et al., 2024). Human-computer interaction can encourage employees’ creative and imaginative behavior, according to research on the relationship between employee innovation and this interaction (Wang and Zhang, 2025). However, previous studies have primarily focused on how employees passively embrace and adapt to technological change, without adopting a micro-level psychological perspective to examine employees’ underlying emotional changes. This study adopts a psychological perspective to examine the basic pathways and mechanisms via which human-computer interaction affects employee creativity.

Conservation of resources (COR) theory posits that psychological resources, which include important psychological components like self-efficacy, psychological safety, and emotional regulation skills, are an essential part of personal resources. Individuals are fundamentally motivated to continuously obtain, preserve, and protect these resources from loss (Hobfoll, 1989). This theoretical framework provides crucial support for unpacking the underlying mechanism that links psychological resources to employee creativity. Dynamic fluctuations of psychological resources are closely associated with two distinct psychological states of employees, and their interrelationship has a double-edged sword effect. Psychological availability, a critical positive psychological state, tends to emerge from employees’ successful acquisition and accumulation of psychological resources. This condition manifests as an individual’s belief in having abundant psychological resources (Kahn, 1990), which serves as crucial psychological support for creativity: When facing novel job demands, employees with ample psychological resources tend to exhibit greater physical and mental readiness and stronger analytical abilities. They are better at interacting with colleagues, more confident in expressing original ideas, and more likely to put innovative ideas into practice—all of which contribute to higher employee creativity (Binyamin and Carmeli, 2010). Employees may, however, experience a negative psychological state characterized by burnout and depleted energy if their psychological resources are excessively drained. An individual’s psychological energy reserves can be significantly reduced by prolonged loss or threat to psychological resources, such as cumulative negative emotions, career uncertainty, or a high-pressure job (Hobfoll, 1989). In addition to contributing to cognitive impairment, such as rigid thinking, distractibility, and difficulties in the associative and integrative processes necessary for creativity, burnout may also trigger defensive motivation. Employees frequently adopt conservative, low-risk work practices, deliberately avoiding innovative tasks requiring significant mental energy, to prevent further depletion of psychological resources (Bakker and de Vries, 2021). To investigate their dual mediating effects on the relationship between human-computer interaction and employee creativity, this study examines psychological availability and burnout together, focusing on employees’ perceptions and dynamic changes in psychological resources.

Self-determination theory holds that interactions between individuals and their environment can foster creativity. Organizational support can help create psychological stability during this process, reducing anxiety when faced with difficult tasks and potentially facilitating employee creativity (Aldabbas et al., 2023). Similarly, conservation of resources theory suggests that organizational support, as a positive external resource, can help replenish depleted individual resources and may encourage employees to adopt innovative activities (Hobfoll, 2001). Building on these theoretical perspectives and examining the influence of psychological resources on employee creativity, this study further explores the moderating role of perceived organizational support as a boundary condition from an external resource compensation perspective, and also investigates the combined effect of individual and organizational resources on employee creativity.

Based on the preceding analysis, this study constructs a comprehensive model to investigate the dual-effect mechanism of human-computer interaction on employee creativity from the perspective of differentiated psychological perceptions. It systematically examines the dual-effect process through which human-computer interaction may influence employee creativity. It emphasizes the distinct moderating role that perceived organizational support plays in these two pathways, positioning it as a crucial boundary condition. To test the dual mediating effects of psychological availability and burnout, as well as the moderating role of perceived organizational support, empirical validation is conducted with field survey data from digital-intelligence transformation enterprises, through structural equation modeling, regression analysis, and bootstrap analysis.

## 2 Conceptual model and hypothesis development

### 2.1 Human-computer interaction and employee creativity

For organizations to manage environmental uncertainty and improve operational efficiency, digital and intelligent transformation has emerged as a key strategic goal. AI applications have expanded beyond single-function scenarios and now form a technological matrix that spans several business modules. Examples of these applications include intelligent scheduling in production processes, decision support in management tasks, and accurate responses in service delivery. With the growing adoption of digital and intelligent technologies, such as artificial intelligence, within organizations, technology has evolved from traditional tool-like attributes to interactive capabilities. This trend has been accompanied by the development of new working systems (Xie et al., 2021).

The main goal of human-computer interaction is to investigate the interactions between humans and computer systems. It seeks to improve human cognitive efficiency and foster better human-computer collaborative performance through multi-layered information exchange and interactive feedback across perception, reception, cognitive processing, decision-making, and execution. Ultimately, this field promotes a cooperative co-creation paradigm that integrates social sustainability with the natural characteristics of interaction (Desolda et al., 2024). Automation and augmentation are two general terms used to describe the historically tool-oriented use of artificial intelligence within organizations. While augmentation refers to close human-computer collaboration to carry out particular tasks, automation involves machines taking over such tasks (Raisch and Krakowski, 2021).

Traditional AI technologies encompass the two paradigms of automation and augmentation, which provide a fundamental theoretical foundation for categorizing different kinds of human-computer interactions. Human-computer interaction can be further classified, based on human-computer role relationships within specific work systems, into competitive AI and collaborative AI. Competitive AI is built on “substitution and surpassing” (Murray et al., 2021), aiming to reach or exceed human performance and ultimately replace human roles. It is characterized by technology taking over individual roles, autonomous AI decision-making, substantially reduced human involvement after decision rights are delegated, and weak perceived accountability for AI outcomes, positioning humans as supervisors (Jarrahi et al., 2021). Collaborative AI, in contrast, is built on “augmentation and empowerment” (Murray et al., 2021). Instead of replacement, it amplifies human effectiveness through complementary capabilities. It features active human participation in decisions, AI-provided decision support, complementarity between AI computation and human experience and intuition, and humans correcting AI biases and errors in close cooperation (Seeber et al., 2020), forming a collaborative pattern of shared goals (Anthony et al., 2023). This distinction is rooted in long-standing concerns about human–system role relationships (Raisch and Krakowski, 2021). Rather than being based on technical functionality, it follows a unifying logic of control authority allocation. Both competitive and collaborative AI originate from the paradigms of automation and augmentation, corresponding to substitution and augmentation agents, and they address the same core question: who leads the task and how decision-making authority is distributed. Competitive AI shifts authority toward the AI, with humans retreating to a supervisory role, embodying substitution and independence. Collaborative AI keeps humans in proactive decision-making roles while AI provides assistance, embodying augmentation and interdependence. Together, they form the two poles of a single human–AI control authority spectrum, jointly covering the full landscape of human–computer interaction in organizational task contexts. They therefore constitute two inseparable sub-dimensions of HCI.

In organizations that increase operational effectiveness, promote product and service innovation, and optimize decision-making quality, long-term competitive advantages tend to be observed. Research has demonstrated an association between human-computer interaction and enhanced organizational effectiveness (Dubey et al., 2019). Accordingly, over 80% of major corporations have incorporated artificial intelligence technologies into their core business processes recently (Dubey et al., 2019).

Employee creativity is increased through human-computer interaction, according to numerous studies (Zang and Li, 2025). This phenomenon is observed when humans work with robots, the robots handle laborious tasks, and humans concentrate on more creative aspects of their jobs. In particular, AI can handle initial procedural tasks while workers concentrate on later phases that involve higher-order problem-solving. This “sequential division of labor” approach is associated with greater employee ingenuity in responding to client inquiries (Jia et al., 2024). Human-computer interaction removes routine responsibilities from employees and increases the overall complexity and difficulty of the issues they face. According to research, such tasks are associated with greater employee drive, proactivity, passion, and entrepreneurial behavior (Amabile and Pratt, 2016; Chae and Choi, 2018; Shin et al., 2017). Additionally, within social networks, AI assistants that possess human-like traits such as anthropomorphism and perceptual intelligence can encourage employee innovation. According to research, perceived anthropomorphism fosters emotive links with AI, while perceptual intelligence fosters instrumental bonds; both kinds of connections foster employee creativity (Zhang et al., 2025).

However, some studies argue that human-computer interaction could stifle employee creativity. Employees are concerned about job displacement due to corporations’ implementation of artificial intelligence (Tong et al., 2021). Major drawbacks of human-computer interaction include information security vulnerabilities, data privacy issues, and disruptive changes triggered by digital transformation. Additionally, employees’ perceptions of job insecurity and the risk of technological displacement further compound these concerns. Taken together, these factors largely explain why employees experience heightened stress and face the risk of technological displacement. When organizations lack managerial commitment and social support, employees’ fear of human-computer interaction and increased job pressure negatively affect their creativity (Hobfoll et al., 2018; Tong et al., 2025).

This study suggests that employee creativity is associated with human-computer interaction in two ways, based on the analysis presented above and the synthesis of viewpoints from different academics. Its particular channels of influence will be examined in the next sections.

### 2.2 The mediating role of psychological availability

An individual’s subjective assessment of the accessibility of resources necessary to complete job tasks-including physical resources, emotional energy, and cognitive abilities-is known as psychological availability. It is an internal psychological state. This subjective experience greatly influences behavioral choices and inclinations (Kahn, 1990). Strength, stamina, and flexibility are examples of physical resources; affective experiences are examples of emotional resources; and pertinent knowledge and skills are examples of cognitive resources (Cai et al., 2018). According to conservation of resources, people actively protect and safeguard resources that will enhance their current or future well-being, while also continuously seeking and creating new resources. In contemporary organizations, artificial intelligence is increasingly being utilized. By automating routine tasks to free up cognitive resources, leveraging intelligent collaboration platforms to provide social support resources, and integrating and optimizing information resources, AI can offer employees significant psychological resources. An individual’s perceived accessibility and the amount of resources they possess influence their psychological availability (Chikoko et al., 2014). As employees recognize the added value of resources generated through human-computer interaction, their evaluations of resource accessibility improve and their psychological availability increases. People with high perceived psychological availability tend to have greater cognitive ability as well as greater emotional and psychological reserves. Individuals in this state of resource abundance are more likely to feel psychologically comfortable and self-sufficient, which boosts their confidence, positive attitudes, and level of involvement at work. Driven by these factors, people are more likely to actively share their knowledge and expertise, put in greater effort, and ultimately achieve synergistic gains in both innovation capacity and productivity (Binyamin and Carmeli, 2010). Accordingly, our study suggests that employees’ perception of human-computer interaction as a means of acquiring more psychological resources is positively associated with their psychological availability and creativity.

We will first examine the association between employees’ psychological availability and competitive and collaborative AI. By easing the strain of mechanical and repetitive tasks, competitive AI relieves employees of monotonous, routine labor and emotionally and physically taxing responsibilities (Vrontis et al., 2023). From the standpoint of emotional resources, traditional, monotonous, and uncreative mechanical jobs generate unpleasant feelings in employees. Consequently, employees must self-regulate their attention and emotions, a process that gradually depletes their limited psychological reserves. Competitive AI frees employees from these constraints. It not only reduces emotional distress and enhances emotional experiences but also provides opportunities for creative work that generates positive emotions. This accumulation of emotional resources, in turn, strengthens employees’ confidence in their resource reserves, potentially enhancing their psychological availability (Russo et al., 2016). From the standpoint of cognitive resources, an individual’s knowledge is represented as a network of interconnected schemata that include related elements such as concepts, facts, and information (Dane, 2010). Collaborative AI can provide employees with the information, skills and knowledge they need to perform their tasks (Xie et al., 2026). When employees are freed from repetitive, cognitively taxing administrative tasks, their cognitive capacity is freed up. Additionally, collaborative AI helps employees discover new interrelationships by revealing hidden links and innovative ways to integrate different knowledge areas. This approach encourages the development of creative ideas and application models by facilitating divergent thinking (Grimes et al., 2023). Based on the above reasoning, the following hypotheses are proposed:

*H1a*. Competitive AI is positively associated with psychological availability.

*H1b*. Collaborative AI is positively associated with psychological availability.

Second, we will elaborate on how psychological availability fosters creativity in employees. There is now widespread agreement based on extensive research on the connection between psychological availability and creative and innovative behavior. On the one hand, high psychological availability provides employees with valuable resources that enhance their creativity and enable them to demonstrate their innovative skills (Wang et al., 2021). On the other hand, increased psychological availability also improves employees’ emotional and cognitive skills, which fosters a sense of security and trust during task completion (Song et al., 2024). In addition to increasing task-specific confidence, job satisfaction, and work engagement, such access also facilitates effective goal attainment, which in turn enhances individual creativity and productivity (Łaba and Geldenhuys, 2016). As a result, greater psychological availability encourages employees to approach tasks with a more optimistic outlook, which fosters proactive engagement and creativity. Based on the above reasoning, we propose the following hypothesis.

*H2*. Psychological availability is positively associated with employee creativity.

*H3*. Psychological availability mediates the relationship between human-computer interaction and employee creativity.

### 2.3 The mediating role of burnout

A major area of study in psychology is burnout, which is generally defined as a condition in which a person’s psychological reserves gradually run out after extended exposure to high-stress situations. Emotional exhaustion, depersonalization, and diminished personal accomplishment are the three main components of this concept, which is also known in academia as the three-factor model of burnout (Maslach and Leiter, 2016). According to the conservation of resources theory, a person’s primary behavioral drive manifests itself in both the efficient maintenance of current resources to prevent depletion and the acquisition and development of new ones. Furthermore, the detrimental impacts of resource depletion on a person’s mental health and behavioral patterns that greatly exceed the benefits of comparable levels of resource acquisition (Hobfoll et al., 2018). Human-computer interaction undoubtedly plays a significant role in modern workplaces. In the short term, it replaces tedious core tasks while effectively integrating and optimizing a variety of information resources. In theory, such activity allows employees to focus on more creative tasks by freeing up cognitive resources. The perception of artificial intelligence substitution brought on by human-computer interaction, however, not only encroaches on employees’ cognitive resources but also worsens the depletion of psychological resources from a long-term developmental perspective (Kong et al., 2021). Employee loneliness at work may worsen, which could result in resource attrition and negatively impact social networks (Mu et al., 2023). These negative emotions build up over time, creating psychological pressure that eventually depletes psychological resources in a variety of areas, such as emotional state and self-efficacy. Employees’ burnout levels significantly rise when they think that human-computer interaction is consuming psychological resources. Additionally, workers who are highly psychologically exhausted show signs of decreased inventiveness, decreased excitement, and decreased work drive. In conclusion, our study suggests that employees’ burnout increases is negatively associated with their creativity when they think that their psychological resources are being depleted by human-computer interaction.

We begin by explaining why competitive and collaborative AI exacerbate employees’ burnout levels. From the perspective of emotional exhaustion, employees may encounter job insecurity within the competitive AI interaction framework if they fear that artificial intelligence will supplant them in their positions (Brougham and Haar, 2018). Such feelings can lead to AI anxiety (Pflügner et al., 2021). Employees may become mired in emotional rumination and exhaust their psychological and emotional reserves when they suffer from AI anxiety and negative self-evaluation (Chunping and Xin, 2025). From a depersonalization standpoint, interactions with intelligent systems frequently fall short of offering emotional support or a sense of interpersonal connection, even though collaborative AI can help employees complete tasks (Tang et al., 2023). According to current research, human psychological well-being is fundamentally dependent on secure, positive, and long-lasting interpersonal relationships (Baumeister and Leary, 1995). When employees and computer interact, a lack of communication in the workplace can lead to negative emotions such as loneliness, which in turn drains psychological resources. From the standpoint of personal accomplishment, the use of collaborative AI boosts output, modifies production processes, and encourages the reorganization of task structures within current positions (Bloom et al., 2018). This shift not only elevates the standard of work (He and Qiu, 2020), but also increases the pressure on employees’ knowledge bases and skill sets to adapt to increasingly complex work environments (Kopytov et al., 2018). As a result, employees may perform worse because they are unfamiliar with new tasks and need to acquire new skills. According to qualitative research using a sample of electronic R&D engineers, the integration of AI technology has significantly increased the skill requirements for software programming operations among junior and intermediate engineers. Engineers experience decreased satisfaction and a diminished sense of accomplishment as a result of work fatigue. Employees may view human-computer interaction as a barrier that depletes psychological resources as a result of these factors, which in turn increases their levels of burnout. Based on the above reasoning, the following hypotheses are proposed:

*H4a*. Competitive AI is positively associated with burnout.

*H4b*. Collaborative AI is positively associated with burnout.

Second, we examine the association between burnout and employee creativity. Creativity is recognized in academia as an essential source of competitive advantage for businesses, as well as a fundamental prerequisite for organizational innovation (Amabile and Pratt, 2016). As a result, management scholars have extensively recognized the importance of employee creativity and studied its antecedents in great detail, both empirically and theoretically. Many studies concentrate on individual-level factors, especially the important impact that emotional states have on creative performance. Research indicates that negative emotional states considerably impair the ability to think creatively (Zhang et al., 2019). Furthermore, individuals experiencing high levels of burnout at work are less likely to generate original ideas and exhibit novel behaviors. Notably, emotional and cognitive depletion frequently accompany burnout. When employees feel that their psychological resources are being depleted, they are therefore more likely to experience negative emotions like exhaustion and irritation, further inhibiting their creativity (Bunjak et al., 2021). Based on the above reasoning, the following hypothesis is proposed:

*H5*. Burnout is negatively associated with employee creativity.

*H6*. Burnout mediates the relationship between human-computer interaction and employee creativity.

### 2.4 The moderating role of organizational support perception

Perceived organizational support refers to employees’ perceptions of the extent to which the company values their contributions and cares about their well-being. Perceived organizational support fundamentally signals that the organization respects, empowers, and safeguards the rights of its employees (Eisenberger et al., 1986), thereby embodying the emotional support employees receive. When organizations express gratitude, provide recognition, and offer support to employees, this can boost their sense of self-efficacy (LIU et al., 2019), thereby enhancing their confidence in achieving their goals. Additionally, organizational support also encompasses instrumental resources that an organization provides to facilitate employees’ work, such as information, training, and material equipment (McMillan, 1997). Accordingly, resources originating from the organization can be considered a form of perceived organizational support.

By helping employees disengage from emotionally and physically taxing professional duties, human-computer interaction enables employees to conserve resources such as time and emotional reserves, thereby promoting positive psychological states. Perceived organizational support greatly increases employees’ internal resource reserves, serving as an essential mechanism that supplements external resources (Nui et al., 2024). When employees have greater resources, they frequently use those resources for creative endeavors, which increases their creativity (Song et al., 2025). Additionally, a strong sense of organizational support helps employees understand the meaning and value of their work by enabling them to tangibly perceive the organization’s respect and recognition (Li et al., 2020). With the help of external resources, employees adopt proactive work attitudes driven by intrinsic motivation, which encourages them to actively participate in creative endeavors and eventually enhance their creativity.

Employees who receive more organizational support are more confident in their ability to overcome resource scarcity constraints, which helps them mitigate and regulate negative emotions in human-computer interaction work contexts where they perceive potential threats from AI applications and feel inadequately equipped to cope (Nui et al., 2024). When employees experience burnout, the resulting psychological resource depletion highlights the importance of resource acquisition. Resources acquired during the depletion process frequently exhibit stronger positive motivating effects (Hobfoll et al., 2018). On the one hand, organizational support—such as equipment provision, task assistance, managerial empathy, and peer mutual aid—can fill resource gaps when employees suffer from cognitive resource depletion and emotional energy exhaustion as a result of burnout. This lowers barriers to creative behavior and fosters creativity by allowing employees to retain the emotional and cognitive flexibility needed for creative endeavors (Lin et al., 2022). On the other hand, with long-term organizational assistance—including career development training and fair incentive programs—employees can accumulate resources and build a “resource safety cushion” (Hong et al., 2024). This effectively encourages work engagement and creativity by enabling them to take on creative tasks even when experiencing fatigue. Based on the above reasoning, the following hypothesis is proposed:

*H7*. The strength of the positive association between psychological availability and employee creativity varies with the level of perceived organizational support. At the same time, perceived organizational support significantly moderates the mediating effect of psychological availability on the relationship between human-computer interaction and employee creativity, indicating a moderated mediation effect.

*H8*. The strength of the negative association between burnout and employee creativity varies with the level of perceived organizational support. At the same time, perceived organizational support significantly moderates the mediating effect of burnout on the relationship between human-computer interaction and employee creativity, indicating a moderated mediation effect.

**Figure 1:**
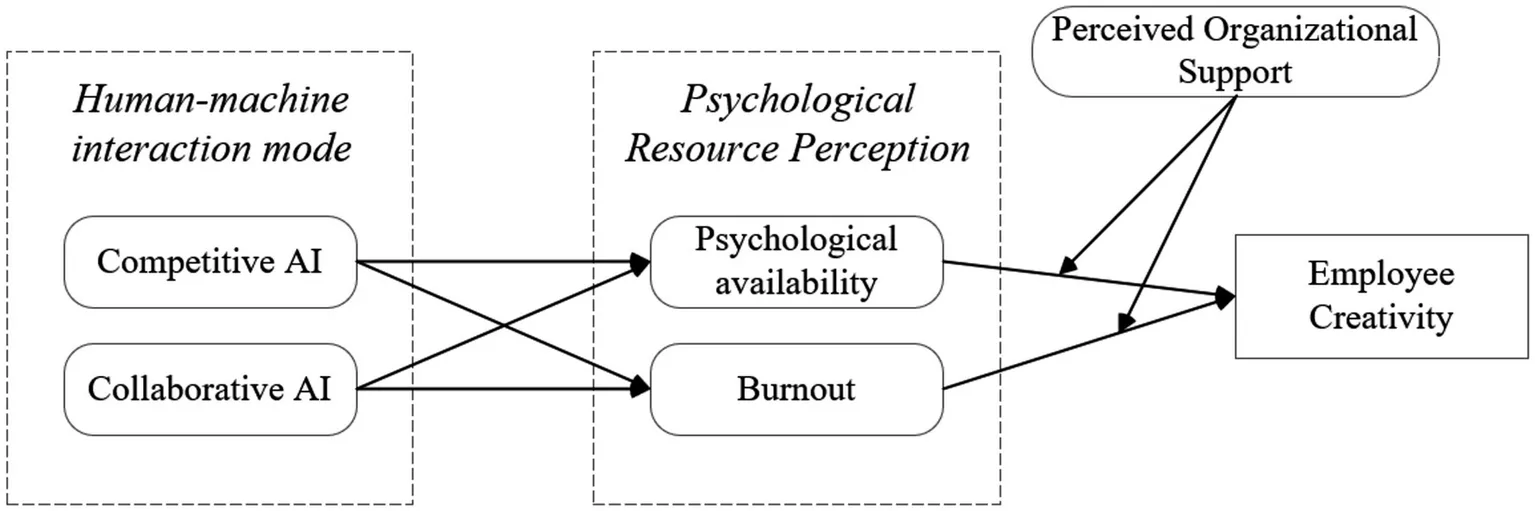
Theoretical model.

In conclusion, Figure 1 illustrates the theoretical model developed for this investigation.

## 3 Research design

### 3.1 Research sample and data collection

This study focuses on businesses undergoing digital and intelligent transformation, with selection criteria requiring employees to have used AI technology and related tools during their employment. In addition to having a thorough comprehension of basic AI theories, respondents must have used these technologies in real-world business settings and produced verifiable work feedback and experience summaries. Between August and November 2025, data were collected using a professional questionnaire platform and a targeted sampling strategy. The selected company samples were widely dispersed across major domestic economic centers, including Beijing, Shanghai, Guangzhou, and Xi’an. The study used a dual screening mechanism through the professional platform to ensure sample representativeness and typicality. The first screening required respondents’ roles to involve the deployment of AI technology, and the second screening confirmed their direct involvement in relevant technology application projects. This design greatly improved the accuracy of data collection, thereby enhancing the practical implications of the research findings. Of the 400 returned questionnaires, 33 were declared invalid after thorough screening because they had logical flaws, were missing key information, or were incomplete. This procedure resulted in 367 valid responses, yielding a valid response rate of 91.75%. Table 1 describes the sample’s demographic distribution in depth and offers a solid quantitative basis for subsequent analysis.

**Table 1:** Sample information statistics.

Category	Classification	Frequency	Percentage
Gender	Man	188	51.2
	Woman	179	48.8
Age	20–25	75	20.4
	26–30	128	34.9
	30–35	113	30.8
	36–40	38	10.4
	41 years old and above	13	3.5
Education	Junior college and below	82	22.3
	Undergraduate	241	65.7
	Postgraduate	31	8.4
	Doctorate and above	13	3.5
Tenure	One year and below	54	14.7
	2–3	101	27.5
	4–6	159	43.3
	7–10	37	10.1
	11 years and above	16	4.4

### 3.2 Variable measurement

During the scale development process, this study systematically adopted validated instruments published in internationally recognized journals to ensure the academic rigor and practical utility of the measurement tools. The items were refined through multiple rounds of a translation–back-translation procedure to preserve the core elements of the original theoretical constructs while aligning with the management realities of Chinese enterprises undergoing digital and intelligent transformation. Regarding the questionnaire structure, core variables were measured using a standard five-point Likert scale (1 = strongly disagree, 2 = disagree, 3 = neutral, 4 = agree, 5 = strongly agree), and a demographic information module was also included.

#### 3.2.1 Human-computer interaction mode

The measurement of human-computer interaction was adapted from the AI automation and augmentation frameworks developed by Leyer and Schneider (2021) and Raisch and Krakowski (2021), with items modified to fit the context of the current study. The competitive AI (CompAI) scale consists of six items, including “The application of AI implies we must replace our roles in the workplace with advanced technology.” The collaborative AI (CollabAI) scale comprises five items, such as “We remain actively involved in decision-making processes.”

#### 3.2.2 Psychological availability (PA)

Psychological availability was measured using a five-item scale developed by May et al. (2004). A sample item is “I feel confident in managing conflicting demands at work.”

#### 3.2.3 Burnout (BO)

Burnout was measured using the revised MBI-GS scale developed by Li (2003). Based on the original dimensions, the items within each dimension were adapted to fit the research context. The final scale comprises 12 items across three dimensions. Specifically, the emotional exhaustion(EE) dimension consists of four items, such as “I feel used up at the end of the workday”; the depersonalization(DP) dimension consists of four items, such as “I have become more callous toward people since I took this job”; and the personal accomplishment(PeA) dimension consists of four items, such as “I deal very effectively with the problems of my recipients.”

#### 3.2.4 Perceived organizational support (POS)

Perceived organizational support was measured using the six-item scale developed by Eisenberger et al. (2001). A sample item is “When I make requests, the organization makes every effort to accommodate me.”

#### 3.2.5 Employee creativity (EC)

Employee creativity was measured using the four-item scale developed by Tierney and Farmer (2004). A sample item is “When solving problems, I first try new ideas and approaches.”

## 4 Empirical analysis

### 4.1 Common method bias and reliability and validity testing

To effectively circumvent common method variance (CMV) that may arise from questionnaire surveys, this study first employed procedural controls to reduce CMV at the source. Specifically, to minimize social desirability bias, we implemented procedural controls that included guaranteeing respondent anonymity and explicitly stating in the instructions that no responses were considered correct or incorrect. Building on these procedures, a triple-validation approach was used to systematically evaluate CMV, successfully avoiding any CMV bias resulting from the survey. First, Harman’s single-factor test was conducted on the full sample. The results revealed that the largest factor accounted for 24.644% of the variance, which is far below the 50% critical threshold. Second, a single-factor confirmatory factor analysis was performed for cross-validation, yielding the following model fit indices: *χ*^2^/df = 8.645, RMSEA = 0.145, IFI = 0.439, TLI = 0.405, and CFI = 0.437. All of these indices were substantially worse than those of the theoretical benchmark model. Finally, the unmeasured latent method factor (ULMC) technique was applied. After incorporating the method factor into the model, the fit indices were as follows: *χ*^2^/df = 1.188, RMSEA = 0.023, IFI = 0.987, TLI = 0.985, and CFI = 0.987. Compared with the baseline model that did not include the method factor, the differences in IFI, TLI, CFI, and RMSEA were all less than 0.05, indicating that model fit did not improve substantially. Based on a comprehensive synthesis of these test results, it can be concluded that there is no significant common method variance in the sample data used for this investigation.

Validation were conducted using SPSS 27.0 and Amos 26.0 during the reliability and validity assessment of the measurement scales. Tables 2, 3 present the results in detail. In terms of validity, all standardized factor loadings were above 0.7, and all average variance extracted (AVE) values exceeded 0.5; in terms of reliability, Cronbach’s *α* coefficients for all latent variables exceeded the 0.8 threshold, indicating high internal consistency of the scales. In addition, the composite reliability (CR) values of all constructs exceeded 0.8. All of these results support the strong convergent validity of the measures. To further examine discriminant validity, a series of nested structural equation models were specified and compared using confirmatory factor analysis. The results indicated that the six-factor model demonstrated the best fit and significant discriminant validity among the factors.

**Table 2:** Reliability and validity analysis.

Measurement dimension	Measurement items	Factor loadings	Cronbach’s α	CR	AVE
Competitive AI	CompAI1	0.789	0.900	0.900	0.600
	CompAI2	0.757			
	CompAI3	0.770			
	CompAI4	0.795			
	CompAI5	0.760			
	CompAI6	0.774			
Collaborative AI	CollabAI1	0.769	0.879	0.879	0.593
	CollabAI2	0.759			
	CollabAI3	0.777			
	CollabAI4	0.774			
	CollabAI5	0.770			
Psychological availability	PA1	0.788	0.889	0.889	0.616
	PA2	0.780			
	PA3	0.806			
	PA4	0.791			
	PA5	0.758			
Burnout	EE1	0.803	0.960	0.960	0.669
	EE2	0.839			
	EE3	0.822			
	EE4	0.823			
	PeA1	0.806			
	PeA2	0.798			
	PeA3	0.828			
	PeA4	0.840			
	DP1	0.800			
	DP2	0.841			
	DP3	0.814			
	DP4	0.801			
Perceived organizational support	POS1	0.744	0.881	0.881	0.552
	POS2	0.741			
	POS3	0.752			
	POS4	0.736			
	POS5	0.724			
	POS6	0.761			
Employee creativity	EC1	0.802	0.868	0.869	0.625
	EC2	0.736			
	EC3	0.803			
	EC4	0.819			

**Table 3:** Results of the confirmatory factor analysis.

Model	χ^2^/df	IFI	TLI	CFI	RMSEA
ULMC model	1.188	0.987	0.985	0.987	0.023
Six-factor model	1.233	0.983	0.982	0.983	0.025
Five-factor model	2.125	0.919	0.912	0.918	0.055
Four-factor mode	3.105	0.847	0.836	0.846	0.076
Three-factor model	5.381	0.680	0.659	0.679	0.109
Two-factor model	6.522	0.596	0.570	0.594	0.123
One-factor model	8.645	0.439	0.405	0.437	0.145

### 4.2 Descriptive statistics and correlation analysis

Descriptive statistics and correlation analyses were performed on the survey data using SPSS 27.0. The means of the main study variables were as follows: 3.417 for competitive AI, 3.366 for collaborative AI, 3.317 for psychological availability, 3.419 for burnout, 3.582 for perceived organizational support, and 3.337 for employee creativity.

The results revealed that competitive AI was strongly and positively associated with both burnout and psychological availability. Likewise, psychological availability and burnout were both strongly and positively correlated with collaborative AI. Additionally, the results indicated that employee creativity was significantly positively correlated with psychological availability, whereas burnout was significantly negatively correlated with employee creativity. These correlation results provide a preliminary basis for further empirical testing. Table 4 presents the detailed descriptive statistics and correlation matrix.

**Table 4:** Descriptive statistics and correlation analysis.

Variables	1	2	3	4	5	6	7	8	9	10
1. Gender	1									
2. Age	0.060	1								
3. Education	−0.023	0.006	1							
4. Tenure	0.095	0.463^**^	0.100	1						
5. CompAI	0.030	−0.098	−0.078	−0.020	1					
6. CollabAI	0.051	−0.019	−0.074	0.035	0.461^**^	1				
7. PA	−0.017	−0.010	0.007	0.029	0.344^**^	0.368^**^	1			
8. BO	0.033	−0.032	−0.085	0.009	0.258^**^	0.241^**^	−0.126^*^	1		
9. POS	−0.040	−0.024	0.002	0.027	0.321^**^	0.327^**^	0.416^**^	−0.204^**^	1	
10. EC	−0.031	−0.021	−0.016	0.003	0.375^**^	0.398^**^	0.477^**^	−0.154^**^	0.612^**^	1
M	1.49	2.42	1.93	2.62	3.417	3.366	3.317	3.419	3.582	3.337
SD	0.501	1.037	0.668	0.998	1.04	1.052	1.077	1.112	0.948	1.098

*p < 0.05; **p < 0.01; ***p < 0.001.

### 4.3 Hypothesis testing

#### 4.3.1 Direct effect testing

**Figure 2:**
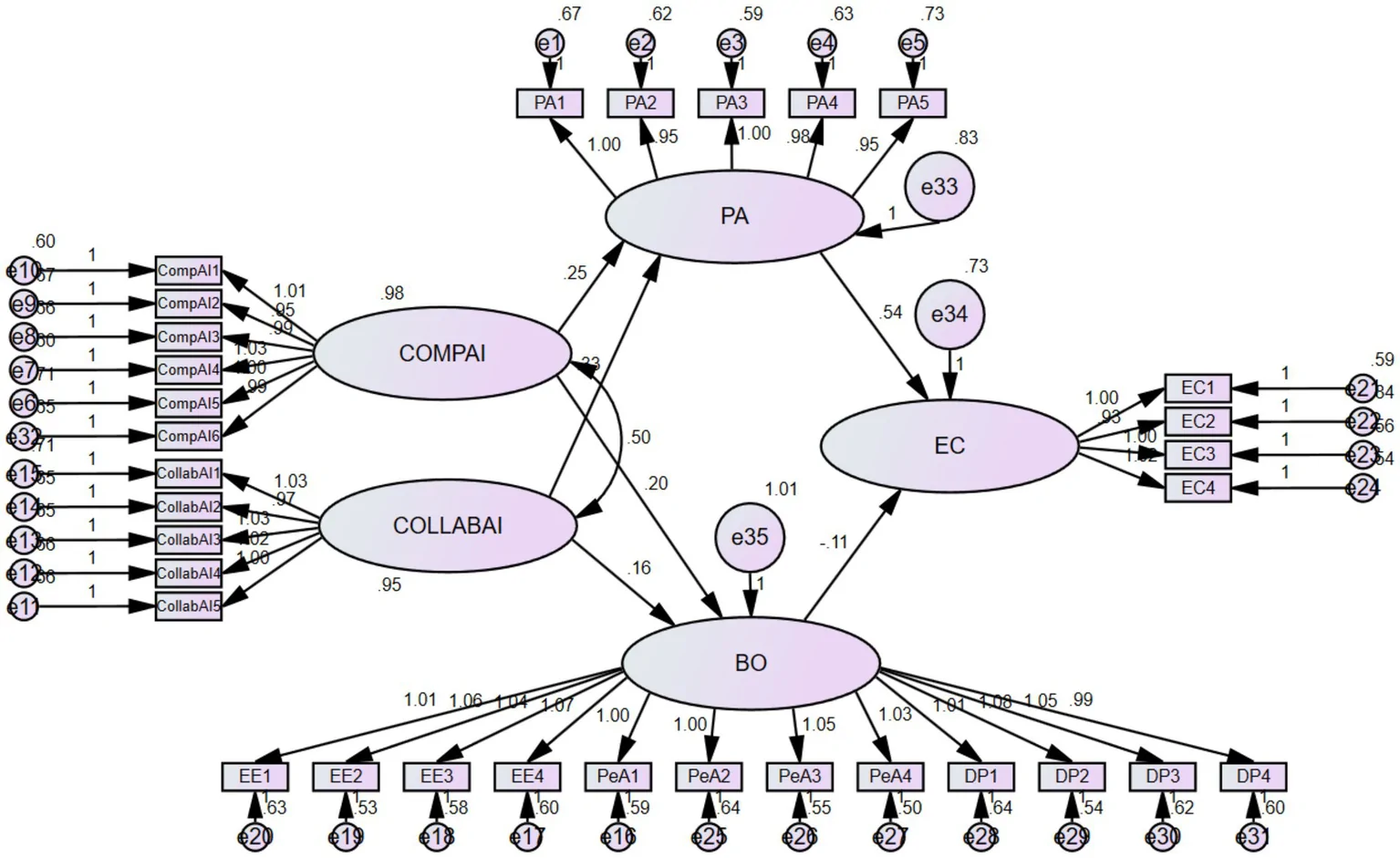
Latent variable path analysis causal model.

This study employed structural equation modeling to empirically test the direct associations among the core variables, incorporating employee gender, age, educational attainment, and years of service as control variables. To prevent interpretive bias, the hypothesized path model from CompAI/CollabAI through psychological availability/burnout to employee creativity should be visualized, clearly displaying the direct relationships and making the linkages among latent variables easy to interpret. To examine the direct paths from competitive AI and collaborative AI to psychological availability and burnout, as well as those from psychological availability and burnout to employee creativity, a structural equation model was built in Amos, as shown in Figure 2.

The most critical finding of this study is that both competitive AI and collaborative AI play a dual role involving resource gain and resource loss: they were positively associated with psychological availability (*γ* = 0.240, *p* < 0.001; *γ* = 0.307, *p* < 0.001) and, simultaneously, with burnout (*γ* = 0.192, *p* < 0.01; *γ* = 0.147, *p* < 0.05). This pattern suggests that human-computer interaction represents a novel context that is linked to two opposing psychological processes at the same time, rather than functioning as a purely positive or negative factor. Competitive AI, through ranking and social comparison, is associated with mastery motivation and competence feedback, which are positively related to psychological availability, while also being tied to persistent comparison pressure that may drain psychological resources. Collaborative AI, although accompanied by resource expansion via task sharing and immediate support, also carries hidden costs such as communication load and role ambiguity. This coexistence of gain and loss pathways indicates that both types of AI are concurrently linked to resource gain and resource loss, and the net outcome is likely to depend on boundary conditions such as interaction intensity, individual differences, and organizational support. These findings offer important theoretical insights into the complex psychological processes that characterize human-AI collaboration (Table 5).

**Table 5:** Results of direct effect structural equation testing.

Independent variables	Dependent variables		
	EC	PA	BO
CompAI	–	0.240^***^	0.192^**^
CollabAI	–	0.307^***^	0.147^*^
PA	0.550^***^	–	–
BO	−0.114^*^	–	–

*p < 0.05; **p < 0.01; ***p < 0.001.

#### 4.3.2 Testing for mediation effects

With 5,000 repeated samples, this study used bootstrap sampling to empirically analyze the significance of the mediating effects. The results revealed that both competitive AI and collaborative AI were indirectly associated with employee creativity through psychological availability and burnout, yet the theoretical mechanisms underlying these associations differed fundamentally. In terms of the data, the indirect effect coefficient of competitive AI via psychological availability was 0.143, with a 95% confidence interval that did not include zero, and the indirect coefficient via burnout was −0.073, with a confidence interval also excluding zero. The corresponding coefficients for collaborative AI were 0.147 and −0.067, both confidence intervals excluding zero. These results suggest that both types of AI were linked to two opposing mediating pathways simultaneously.

Further analysis suggests distinct logics behind these indirect associations. Competitive AI, rooted in substitution, is linked to a positive pathway through emotional resource release when tasks are taken over, which is associated with higher psychological availability. Its negative pathway is tied to perceived substitutability, threatening professional self-concept and representing a psychological drain. Collaborative AI, rooted in augmentation, is associated with a positive pathway through expanded capability boundaries and enhanced psychological availability. Its negative pathway stems not merely from coordination costs but from a deficit in social–emotional connection. Prolonged collaboration with AI replaces interpersonal emotional exchange, limiting access to emotional support and relational belonging. The resulting sense of loneliness constitutes a unique source of burnout in collaborative contexts. Hence, competitive AI is associated with dual pathways of “liberation” and “threat,” while collaborative AI involves “empowerment” and “emotional isolation.” These findings suggest that although the indirect pathways appear similar in form, the underlying mechanisms diverge fundamentally, offering a nuanced lens on the complex aftereffects of human–computer interaction (Table 6).

**Table 6:** Bootstrap mediation effect test results.

Independent variables	Mediator	Dependent variables	Effect value	SE	95% CI	
					LL	UL
CompAI	PA	EC	0.143	0.031	0.086	0.208
CollabAI	PA	EC	0.147	0.031	0.093	0.213
CompAI	BO	EC	−0.073	0.02	−0.115	−0.038
CollabAI	BO	EC	−0.067	0.018	−0.105	−0.034

#### 4.3.3 Moderation effect test

**Figure 3:**
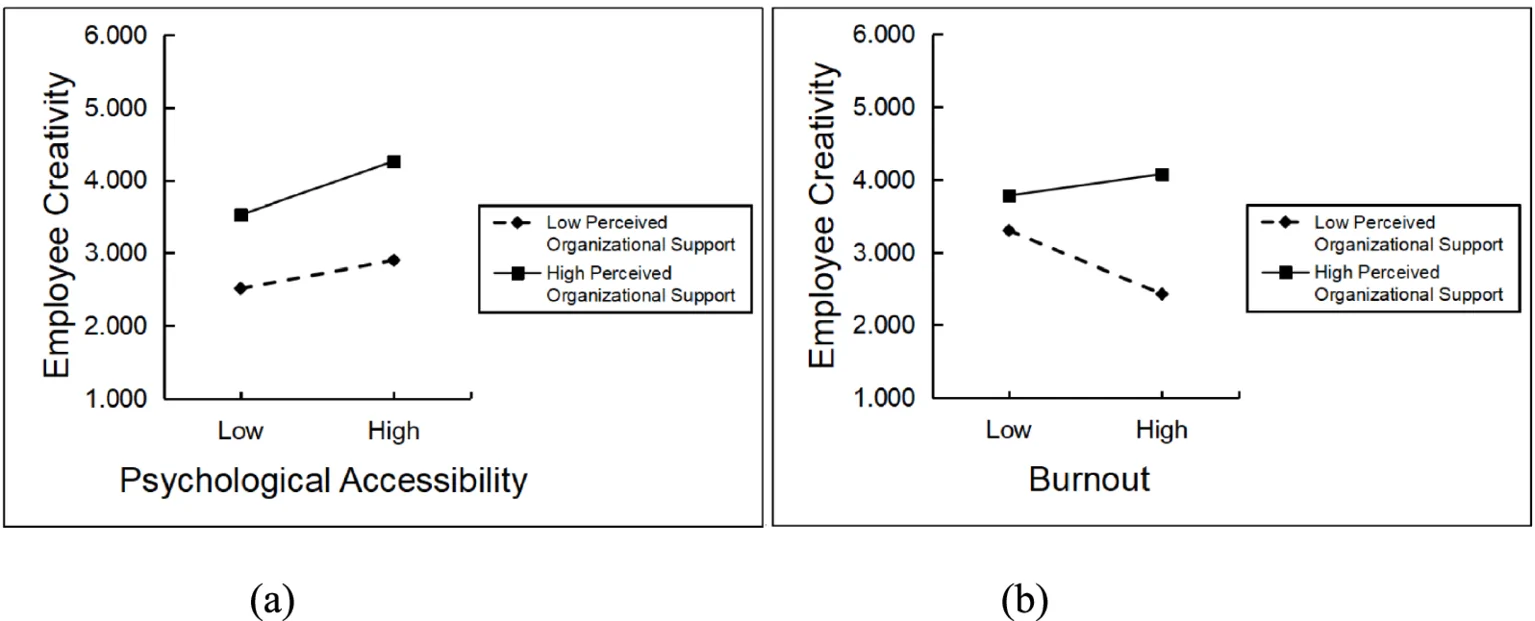
**(a)** The moderating effect of perceived organizational support on the relationship between psychological availability and employee creativity. **(b)** The moderating effect of perceived organizational support on the relationship between burnout and employee creativity.

Hierarchical regression analysis and SPSS 27.0 statistical software were used in this study to examine the moderating role of perceived organizational support in the relationships among the relevant variables. Variables including psychological availability, burnout, and perceived organizational support were mean-centered before the analysis. Subsequently, corresponding interaction terms were created to conduct hierarchical regression analysis; Table 7 presents the detailed results. In terms of model development, M1 served as the baseline model with only control variables to investigate their associations with employee creativity. M2 added psychological availability to M1, and the findings showed that psychological availability was significantly positively associated with employee creativity (*β* = 0.477, *p* < 0.001). M3 further included perceived organizational support, and its regression coefficient was statistically significant (*β* = 0.500, *p* < 0.001). M4 added the interaction term between psychological availability and perceived organizational support to M3, yielding a significant interaction coefficient (*β* = 0.090, *p* < 0.05). Similarly, in the analysis of burnout, M5 added burnout to M1, and the results showed that burnout had a significant negative association with employee creativity (*β* = −0.157, *p* < 0.01); M6 added perceived organizational support to M5, and its regression coefficient was significant (*β* = 0.606, *p* < 0.001); Model M7 added the interaction term between burnout and perceived organizational support to M6, and the interaction coefficient was significant (*β* = 0.245, *p* < 0.001). The results indicated that the interaction term between psychological availability and perceived organizational support was significant (*β* = 0.090, *p* < 0.05). This suggested that the association between psychological availability and employee creativity was significantly moderated by perceived organizational support. Specifically, the positive association between psychological availability and employee creativity became stronger as perceived organizational support increased, thus supporting Hypothesis H7 (see Figure 3a for the moderation effect). The interaction between burnout and perceived organizational support was significant (*β* = 0.245, *p* < 0.001), indicating that the association between burnout and employee creativity was also significantly moderated by perceived organizational support. In other words, the negative association between burnout and employee creativity was weakened when perceived organizational support was higher, thus preliminarily confirming Hypothesis H8 (see Figure 3b for the specific moderating effect).

**Table 7:** Regression test results for the moderating effects of perceived organizational support on psychological availability/burnout and employee creativity.

Variables	EC						
	M1	M2	M3	M4	M5	M6	M7
Gender	−0.032	−0.023	−0.005	−0.005	−0.027	−0.005	−0.003
Age	−0.029	−0.014	0.002	0.000	−0.037	−0.003	0.007
Education	−0.019	−0.020	−0.018	−0.019	−0.033	−0.019	−0.002
Tenure	0.021	0.000	−0.017	−0.015	0.027	−0.009	−0.015
PA		0.477^***^	0.270^***^	0.256^***^			
BO					−0.157^**^	−0.032	−0.130^**^
POS			0.500^***^	0.541^***^		0.606^***^	0.485^***^
PA × POS				0.090^*^			
BO × POS							0.245^***^
*R* ^2^	−0.009	0.218	0.426	0.431	0.013	0.366	0.404
Δ*R*^2^	0.002	0.227	0.206	0.007	0.024	0.35	0.040
*F*	0.176	21.425	46.236	40.609	1.956	36.174	36.484

*p < 0.05; **p < 0.01; ***p < 0.001.

From a conservation of resources (COR) theory perspective, when employees face cognitive resource depletion and emotional exhaustion linked to burnout, instrumental and emotional support from the organization can fill resource gaps, helping sustain the cognitive flexibility and emotional engagement needed for creative activities, thereby building a “resource safety net” against resource loss. Organizational support received during resource loss tends to be more strongly associated with resource replenishment and is related to a greater capacity to navigate resource constraints when facing potential AI threats, alleviating negative emotions and sustaining creative output. Overall, perceived organizational support plays an asymmetric moderating role in the dual pathways of human–computer interaction: it is linked to a strengthening of the resource gain pathway and a weakening of the resource loss pathway, constituting a key boundary condition for understanding the association between human–AI collaboration and employee creativity.

#### 4.3.4 Test for moderated dual mediating effects

The bootstrap sampling technique was used in this study. Resampling procedures were performed after including control variables such as gender, age, years of service, and educational attainment. Under high and low conditions of perceived organizational support (one standard deviation above the mean, +1SD; and one standard deviation below the mean, −1SD), 5,000 bootstrap iterations were performed to examine the indirect associations through psychological availability and burnout. The specific analytical approach involved examining the confidence intervals of the indirect associations under both high and low perceived organizational support conditions. This allowed for assessing whether the indirect associations were statistically significant, thereby providing evidence for the hypothesized mediation.

The statistical results in Table 8 indicate that perceived organizational support significantly moderated the mediating effect of psychological availability in the relationship between competitive AI and employee creativity (index of moderated mediation = 0.032, 95% CI [0.003, 0.067], excluding zero). Specifically, the indirect effect of psychological availability was significant under both low perceived organizational support (*β* = 0.050, 95% CI [0.002, 0.107]) and high perceived organizational support (*β* = 0.111, 95% CI [0.054, 0.182]), and the difference between the two conditions was statistically significant (*β*diff = 0.061, 95% CI [0.006, 0.127]). These results provided preliminary support for Hypothesis 7. From a theoretical perspective, this suggests that perceived organizational support, as a critical contextual resource, is associated with a strengthening of the resource gain pathway through which psychological availability transfers into employee creativity. When organizational support is perceived to be higher, employees are more likely to channel the cognitive resources released by competitive AI into creative exploration, such that the positive mediating role of psychological availability is more pronounced.

**Table 8:** Test results for the moderated double mediating effect.

Moderator	Indirect effect	95% CI		Index	95% CI	
		LL	UL		LL	UL
Path 1: CompAI-PA-EC						
Low POS(−1SD)	0.050	0.002	0.107	0.032	0.003	0.067
High POS(+1SD)	0.111	0.054	0.182			
Difference	0.061	0.006	0.127			
Path 2: CollabAI-PA-EC						
Low POS(−1SD)	0.051	0.004	0.107	0.032	0.000	0.066
High POS(+1SD)	0.112	0.056	0.179			
Difference	0.061	0.001	0.126			
Path 3: CompAI-BO-EC						
Low POS(−1SD)	−0.108	−0.192	−0.049	0.063	0.025	0.118
High POS(+1SD)	0.011	−0.019	0.046			
Difference	0.119	0.047	0.223			
Path 4: CollabAI-BO-EC						
Low POS(−1SD)	−0.105	−0.188	−0.049	0.061	0.025	0.116
High POS(+1SD)	0.010	−0.016	0.045			
Difference	0.115	0.047	0.220			

Perceived organizational support also moderated the mediating effect of psychological availability in the relationship between collaborative AI and employee creativity (index of moderated mediation = 0.032, 95% CI [0.000, 0.066]). The indirect effect of psychological availability was significant under low perceived organizational support (*β* = 0.051, 95% CI [0.004, 0.107]) and even stronger and significant under high perceived organizational support (*β* = 0.112, 95% CI [0.056, 0.179]), with a statistically significant difference between conditions (*β*diff = 0.061, 95% CI [0.001, 0.126]). This further supports Hypothesis 7. The finding indicates that perceived organizational support not only directly supplements individual resources but also amplifies the indirect relationship between collaborative AI and creativity through enhanced perceptions of resource availability. High perceived organizational support is linked to greater experiences of empowerment and security in human–computer collaboration, which are related to a more complete transformation of augmented capability boundaries into creative output.

With regard to the burnout pathway, perceived organizational support significantly moderated the mediating effect of burnout in the relationship between competitive AI and employee creativity (index of moderated mediation = 0.063, 95% CI [0.025, 0.118]). Under low perceived organizational support, the indirect effect via burnout was significantly negative (*β* = −0.108, 95% CI [−0.192, −0.049]); under high perceived organizational support, this indirect effect was no longer significant (*β* = 0.011, 95% CI [−0.019, 0.046]), and the group difference was statistically significant (*β*diff = 0.119, 95% CI [0.047, 0.223]). These results provided preliminary support for Hypothesis 8. From a conservation of resources perspective, when organizational support is low, the perceived threat of substitutability associated with competitive AI is linked to heightened resource loss, making the negative mediating pathway via burnout more prominent. Conversely, the instrumental and emotional resources provided by high perceived organizational support are linked to effective compensation for the depletion of cognitive and emotional energy, thereby buffering the negative mediating role of burnout and even rendering it non-significant. This reveals the critical buffering function of perceived organizational support within the resource loss pathway.

Similarly, perceived organizational support significantly moderated the mediating effect of burnout in the relationship between collaborative AI and employee creativity (index of moderated mediation = 0.061, 95% CI [0.025, 0.116]). Under low perceived organizational support, the indirect effect was significantly negative (*β* = −0.105, 95% CI [−0.188, −0.049]); under high perceived organizational support, it was not significant (*β* = 0.010, 95% CI [−0.016, 0.045]), and the between-group difference was statistically significant (*β*diff = 0.115, 95% CI [0.047, 0.220]). This supports Hypothesis 8. The finding suggests that perceived organizational support is associated with a weakening of the detrimental mediating role of burnout. In the context of collaborative AI, high perceived organizational support is linked to the alleviation of burnout arising from deficits in social–emotional connection and feelings of loneliness, providing employees with emotional belonging and recognition, and thus helping to protect creativity from depletion. This highlights the emotional compensation function of organizational support in human–computer collaboration and underscores its importance as a “resource safety net” for sustaining innovative vitality.

## 5 Conclusion and discussion

### 5.1 Research findings

The purpose of this study is to examine whether and how employee creativity is associated with human-computer interaction. It investigates the relationship between employee creativity and human-computer interaction, as well as the mediating effects of burnout and psychological availability, using survey data from 367 Chinese corporate employees. Furthermore, the study demonstrates the moderating role of perceived organizational support. The study yields three key findings: (1) This study classifies such interactions into competitive and collaborative AI modes based on human-computer role relationships, explicitly distinguishing this dimensional framework from previous studies and expanding the research perspective on human-computer interaction and resource conservation theory. (2) In contrast to previous findings, this study demonstrates that human-computer interaction is significantly associated with employee creativity. This study constructs both positive and negative pathways, focusing on the dynamic fluctuations in employees’ psychological resources. Through the mediating effect of psychological availability that arises from work, human-computer interaction shows a positive relationship with employee creativity along the positive pathway. Through the mediating effect of burnout arising from work, human-computer interaction is negatively associated with employee creativity along the negative pathway. These results deepen our knowledge of the association between employee creativity and human-computer interaction. (3) There are distinct moderating roles of perceived organizational support. In particular, within the positive pathway, perceived organizational support moderates the mediating effect of psychological availability and is associated with a strengthening of this positive mediating effect. Within the negative pathway, perceived organizational support is associated with a weakening of the negative mediating effect of burnout.

### 5.2 Theoretical contributions

This study draws a clear distinction between competitive and collaborative AI through a more nuanced typology of human-computer interaction patterns grounded in the interplay between human and computer roles. Specialized studies examining employee psychological states within human-computer interaction contexts remain scarce, and the majority of existing research focuses primarily on the application of artificial intelligence technologies and the association between AI’s disruptive effects and employee creativity (Fu and Xu, 2025). This study systematically maps the underlying pathways through which human-computer interaction is associated with employee creativity. The framework for human-computer interaction at the micro-organizational level is strengthened by incorporating a more refined classification of interaction modes, thereby better clarifying the fundamental mechanisms linking these two domains.

This study’s primary analytical lens is the dynamic evolution of employees’ psychological resources, grounded in resource conservation theory. To systematically explain the double-edged sword process through which human-computer interaction is associated with employee creativity, it develops a dual-mediation theoretical framework termed “psychological availability-burnout.” Research to date on AI has largely exhibited a one-sided cognitive bias, focusing primarily on its singular associations with employees. Some studies suggest that AI is positively associated with employee work performance (Sheng et al., 2022), while others indicate that AI is linked to heightened job insecurity among employees (Wang et al., 2019). The academic community has not yet thoroughly and systematically investigated the dual pathways through which human-computer interaction is related to employee creativity. This study establishes a two-pronged theoretical model linking human-computer interaction with employee creativity through the integration of both positive and negative pathways into a single analytical framework. This model allows for a comprehensive examination of the various ways in which human-computer interaction is associated with employee creativity, providing a robust foundation for advancing research in this domain.

To construct a moderated mediation analysis model, this study incorporates perceived organizational support as a moderator variable into the research framework. This study empirically identifies and investigates the differential moderating role of perceived organizational support in the positive and negative pathways linking human-computer interaction with employee creativity: on the one hand, perceived organizational support is associated with a weakening of the negative mediating effect of burnout in this relationship; on the other hand, it is associated with a strengthening of the positive mediating effect of psychological availability in the relationship between human-computer interaction and employee creativity. These results elucidate the logical chain linking these variables and define important boundary conditions for the association between employee creativity and human-computer interaction. Thus, this study opens up new avenues and research directions for investigating boundary effects in the context of employee creativity.

### 5.3 Management implications

Managers are advised to formulate differentiated implementation strategies based on the distinct characteristics of competitive AI and collaborative AI. For competitive AI, a human-computer task suitability assessment should be conducted prior to deployment. Tasks that are highly repetitive and rule-based may be allocated to AI, while those requiring judgment, creativity, or interpersonal nuance may be retained for employees, with role boundaries clearly documented to prevent role ambiguity. In performance feedback sessions, AI should be explicitly positioned as a benchmark that provides reference information rather than as a substitute for human workers; such framing is associated with lower perceived threat among employees. For collaborative AI, standard operating protocols should be formalized. For instance, a workflow consisting of an AI-generated draft, subsequent human refinement, and a final mutual review can be established, with clear division of responsibilities and handover criteria at each stage. Quarterly human-computer collaboration review meetings are recommended to gather employee feedback and continuously improve the collaboration process. Furthermore, case-based training sessions prior to deployment can help communicate the logic and value of each AI mode, which is related to building a shared mental model for effective human-computer interaction.

A regular monitoring and intervention system for employee psychological resources is recommended. Standardized scales measuring psychological availability and burnout may be administered on a quarterly basis, and departmental early-warning ledgers can be created to track trends. For teams exhibiting relatively low psychological availability, task-crafting interventions that grant employees the autonomy to flexibly switch between AI-assisted and autonomous work modes are associated with greater resource replenishment. For burnout, a tiered intervention approach is suggested. Mild burnout may be linked to flexible work hours or short-term job rotation; moderate to higher levels of burnout should be addressed through professional Employee Assistance Program (EAP) counseling, combined with stress management and emotion regulation workshops. A designated human-computer collaboration adaptation period of at least one quarter, during which the depth of AI involvement increases gradually, is associated with a smoother transition and reduced resource depletion. Such a period allows employees to adjust to new workflows without abrupt psychological strain, and is linked to more sustainable integration of AI technologies.

A dual-track organizational support system that integrates instrumental and emotional components is advised. Instrumentally, organizations may establish an “AI Skills Development Fund” to provide each employee with an annual minimum amount of training in areas such as data literacy and human-computer collaboration process management. Innovation incentive programs should explicitly recognize and reward creative outputs generated through human-computer collaboration, reinforcing the link between collaborative efforts and employee creativity. Emotionally, managers are encouraged to hold monthly one-on-one feedback sessions dedicated to employees’ experiences and feelings in human-computer collaboration, focusing on listening rather than evaluating task performance. Honorary awards, such as a “Human-Computer Collaboration Contribution Award,” can be established to acknowledge employees’ active exploration and contributions in intelligent collaboration. An internal knowledge-sharing platform that allows employees to post collaboration stories and coping strategies is associated with the development of a peer support network, which can further strengthen perceived organizational support. The synergy of instrumental and emotional supports is associated with maximizing the positive moderating role of perceived organizational support in the association between human-computer interaction and employee creativity.

### 5.4 Research limitations and future prospects

This study offers avenues for further investigation in the following aspects, though it also has a number of limitations that warrant attention: (1) No comparative empirical research has been conducted across cultures and work positions; the analysis relies solely on cross-sectional data from 367 employees within Chinese digital firms. Given the range of cultural backgrounds, economic development levels, and management styles worldwide, the generalizability of the findings is associated with the distinctiveness of the current sample. Cross-national comparative evaluations are essential, especially given the current widespread adoption of AI globally. (2) In terms of mediating mechanisms, this study focuses primarily on the dynamic evolution of psychological resources, without examining cognitive pathways such as creative thinking and information integration. To advance research on human-computer interaction and employee creativity, future studies could examine additional mediating pathways, such as creative self-efficacy and knowledge-sharing willingness. (3) Currently, this study examines only the mechanisms associated with employee creativity. Future studies could broaden the focus from the individual to the team level, exploring the unique associations of human-computer interaction with team creativity, as well as its characteristic patterns at the team level.

## Data Availability

The original contributions presented in the study are included in the article/supplementary material, further inquiries can be directed to the corresponding author.
